# Putative Breast Cancer Driver Mutations in *TBX3* Cause Impaired Transcriptional Repression

**DOI:** 10.3389/fonc.2015.00244

**Published:** 2015-10-29

**Authors:** Kathrin Fischer, Gert O. Pflugfelder

**Affiliations:** ^1^Institute of Genetics, Mainz University, Mainz, Germany

**Keywords:** *TBX3*, breast cancer, somatic mutations, p*21*, frameshift mutation, in-frame deletion, driver mutation

## Abstract

The closely related T-box transcription factors TBX2 and TBX3 are frequently overexpressed in melanoma and various types of human cancers, in particular, breast cancer. The overexpression of *TBX2* and *TBX3* can have several cellular effects, among them suppression of senescence, promotion of epithelial–mesenchymal transition, and invasive cell motility. In contrast, loss of function of *TBX3* and most other human T-box genes causes developmental haploinsufficiency syndromes. Stephens and colleagues (1), by exome sequencing of breast tumor samples, identified five different mutations in *TBX3*, all affecting the DNA-binding T-domain. One in-frame deletion of a single amino acid, p.N212delN, was observed twice. Due to the clustering of these mutations to the T-domain and for statistical reasons, *TBX3* was inferred to be a driver gene in breast cancer. Since mutations in the T-domain generally cause loss of function and because the tumorigenic action of TBX3 has generally been attributed to overexpression, we determined whether the putative driver mutations had loss- or gain-of-function properties. We tested two in-frame deletions, one missense, and one frameshift mutant protein for DNA-binding *in vitro*, and for target gene repression in cell culture. In addition, we performed an *in silico* analysis of somatic *TBX* mutations in breast cancer, collected in The Cancer Genome Atlas (TCGA). Both the experimental and the *in silico* analysis indicate that the observed mutations predominantly cause loss of TBX3 function.

## Introduction

Cancer is assumed to progress by a type of Darwinian evolution that operates at the cellular rather than the organismic level. Mutations arise in an approximately random fashion. Those that increase the fitness of the affected cell relative to its neighbors (driver mutations) will be positively selected (and *vice versa*). Most mutations are neutral (passenger mutations) or deleterious from the perspective of the cancer cell. New sequencing techniques allow increasingly fast and affordable acquisition of DNA sequence information from cancer tissue, either of the exome or the complete genome. Genes that in a mutated or misexpressed form can promote cancer development are operationally defined as cancer genes. Most cancer mutations act dominantly (oncogenes) indicating gain-of-function, or possibly haploinsufficiency. Less than 10% are assumed to be tumor suppressors, requiring homozygosity of the mutant allele for the tumor promoting effect. The discrimination between driver and passenger mutations is not trivial (2, 3). It is plausible to assume and is born out by the mutation pattern of *bona fide* oncogenes that, in the category of point mutations, only particular missense mutations are able to cause an activated gene product. On the other hand, tumor suppressor genes can be inactivated by a higher proportion of missense mutations, in addition to nonsense and frameshift mutations, in a large part of the protein coding region. Vogelstein and colleagues proposed a rule of the thumb according to which >20% of all mutations in a gene must be recurrent missense mutations in particular positions to qualify it as an oncogene, while a tumor suppressor gene should contain >20% of inactivating mutations (3). It should be added that only mutations in tissues in which these genes are active should be taken into account.

The two closely related T-box transcription factors TBX2 and TBX3 are considered oncogenes because they are frequently overexpressed in melanoma (4–6) and various types of human cancer, such as breast, bladder, liver, and pancreas carcinoma (7–11). These correlative data are supported by direct biological evidence. TBX2/3 can contribute to transformation by suppressing senescence and anoikis (5, 12–16). TBX2/3 can promote epithelial–mesenchymal transition (EMT) and invasive cell behavior in melanoma and breast cancer cells (6, 10, 17, 18) and in heterospecific epithelia (19). TBX2/3 may also contribute to breast cancer growth by promoting proliferation of cancer stem-like cells (20, 21). Part of this may be attributed to the induction of polyploidy and resistance to anti-cancer drugs (cisplatin), which can be caused by TBX2 overexpression (22, 23). TBX2/3 can also stimulate proliferation (17, 21, 24–28). Cell lines overexpressing TBX2 can show TBX2 addiction (24). TBX2/TBX3 represses expression from the long control region (LCR) of human papilloma viruses HPV16 and 18, the main causative agents of cervical cancer. LCR repression is enhanced by interaction of TBX2/3 with the HPV L2 minor capsid protein. L2 and TBX2 were found to colocalize in tissue sections of cervical intraepithelial neoplasia (29).

TBX2 and TBX3 are members of the Tbx2 subfamily of T-box transcription factors (30). Tbx2 (12, 31–34) and Tbx3 (35–37) are predominantly transcriptional repressors. Recently a role of TBX3 (and TBX5) also in splicing has been recognized (38, 39). Tbx2 and Tbx3 are important developmental regulators controlling, among others, the development of heart, kidney, limbs, lung, the visual system, and mammary tissue (8, 40–47). Null mutations in most members of the human Tbx gene family cause heritable haploinsufficiency syndromes. TBX3 haploinsufficiency is the cause of ulnar-mammary syndrome (48, 49). Some of the oncogenic properties of TBX2/3 appear conserved in evolution. In *Drosophila*, the only Tbx2 subfamily gene is *optomotor-blind* (*omb*, FlyBase: *bifid, bi*) (50). Overexpression of *omb* or TBX2 in an epithelium of the developing fly, the wing imaginal disk, causes two types of cellular motility: migration in the plane of the epithelium and basal delamination with penetration of the basal membrane (19). Omb also controls cell proliferation in a context-dependent manner (51, 52).

Although Tbx2 and Tbx3 are often both expressed in the development of a given organ, their expression at tissue and cellular level can diverge in time and space. During mouse embryonic breast development, Tbx2 and Tbx3 are co-expressed in mesenchyme but only Tbx3 is expressed in epithelial tissue (mammary placode and branching mammary duct epithelium). Tbx2 is not expressed in postnatal stages (53), whereas Tbx3 remains expressed throughout breast development (54, 55). Although TBX2 and TBX3 share many properties at the molecular and cell biological level, they can be functionally distinct, at least in melanoma cells (17, 56).

According to current knowledge based on studies in cell culture and experimental animals, the tumorigenic action of TBX2/3 appears predominantly caused by gain-of-function (transcriptional upregulation or gene amplification). This is supported by the finding that TBX2 or TBX3 are overexpressed/amplified in melanoma and various types of cancer and that the degree of overexpression correlates with invasiveness, distant metastasis, and poor prognosis (11, 57–59).

In recent exome analyses of somatic mutations in breast cancer, several mutations in *TBX3* were identified. Stephens and colleagues in an analysis of 350 breast cancer samples found three short deletions (in each case resulting in the in-frame deletion of one amino acid; N212 was deleted in two cancers) and three truncating mutations. All changes clustered within 50 amino acids of the TBX3 T-domain. Based on the high relative mutation frequency and the apparent non-random clustering of the mutation sites, *TBX3* was considered a driver gene (1). Due to the recurrence of ΔN212, *TBX3* also fulfils the driver gene definition of Vogelstein et al. (3). Kandoth et al. analyzed the exome of 12 tumor types present in The Cancer Genome Atlas (TCGA). Using the MuSIC tool (60) they identified *TBX3* as “significantly mutated gene” (SMG) with an increased mutation rate relative to background, in particular in breast cancer. ΔN212 was also identified in their analysis of 763 breast cancer specimen (61).

We here investigate the molecular function of four putative driver mutations identified by Stephens et al. (1) and Kandoth et al. (61) in order to determine whether they have gain- or loss-of-function properties *in vitro* and *in vivo*. We, furthermore, analyze the occurrence and distribution of *TBX3* mutations in breast cancer in relation to other *TBX* genes and to other tumors.

## Materials and Methods

### Protein Sequence Alignment

Human TBX protein sequences were aligned with Clustal Omega (62). The following sequences were used (gene name and Ensembl protein number): EOMES ENST00000449599, T ENST00000296946, TBR1 ENST00000389554, TBX1 ENST00000332710, TBX2 ENST00000240328, TBX3 ENST00000349155, TBX4, ENST00000240335, TBX5 ENST00000310346, TBX6 ENST00000395224, TBX10 ENST00000335385, TBX15 ENST00000207157, TBX18 ENST00000369663, TBX19 ENST00000367821, TBX20 ENST00000408931, TBX21 ENST00000177694, TBX22 ENST00000373294.

### Plasmid Constructions

Primer sequences are provided in Table S5 in Supplementary Material. All unmodified oligonucleotides were synthesized by Sigma-Aldrich, Taufkirchen, Germany.

Construction of cell culture TBX3 expression clones: starting clone TBX3-3xFLAG/pENTR/D-TOPO was constructed by A. Legler based on a TBX3 + 2a cDNA clone (ImaGenes, Berlin, Germany). Mutations were introduced using the QuikChange^®^ II XL Site-Directed Mutagenesis Kit (Agilent, Waldbronn, Germany) and primers 1379/1380 for TBX3dm (G129A/R130S), 1562/1563 for Y163fs2*, 1784/1785 for H187Y, 1564/1565 for T210delT, and 1560/1561 for N212delN. The C-terminally FLAG-tagged sequences were transferred to pT-Rex-DEST30 using Gateway^®^ LR Clonase™ II Enzyme Mix (Invitrogen, Darmstadt, Germany).

Construction of bacterial expression clones: DNA encoding the first 342 amino acids of TBX3 (normal or mutant sequence) were amplified by linker PCR from the corresponding TBX3-3xFLAG/pENTR/D-TOPO clones. Primers 1879 and 1880 containing *Bam*HI and *Sal*I restriction sites, respectively, were used for amplification. These sites were used to clone the amplificate into pGEX-2TK (GE Healthcare).

Construction of p21-FLuc (firefly luciferase) expression vector: 2335 bp from the promoter region of the *p21* gene (*CDKN1A*, ENSG00000124762) were amplified from a genomic clone (obtained from G. Spoden) by linker PCR using primers 1808 and 1323. The *SV40* promoter was removed from the pGL3-control vector (Promega, Mannheim, Germany) by *Kpn*I/*Hin*dIII digest and replaced by the *p21* promoter fragment by T4 DNA ligase ligation.

### Bacterial Expression and Purification of TBX3 Mutant Proteins

GST-TBX3(1–342) in pGEX-2TK and its mutant derivates were expressed in *Escherichia coli BL21DE3* (New England Biolabs) by isopropyl-β-d-thiogalactopyranosid (IPTG) induction. Cleared sonified cell lysate was chromatographed on glutathione agarose (Cube Biotech, Monheim, Germany) following the company’s instructions^1^. Protein concentration in eluate fractions were measured by Bradford assay (Roti-Quant, Carl Roth, Karlsruhe, Germany).

### Electrophoretic Mobility Shift Assay of GST-TBX3 Protein Mutants

Electrophoretic mobility shift assays were performed using a double-stranded 5′ digoxigenin (DIG)-labeled 24 base oligonucleotide (AATTTCACACCT AGGTGTGAAATT) containing the Brachyury consensus target site (synthesized by MWG Biotech/Eurofins, Ebersberg, Germany). GST-tagged proteins were incubated with DIG-oligo at 15°C for 1 h in electrophoretic mobility shift assay (EMSA) buffer [20 mM HEPES, 100 mM KCl, 1 mM DTT, 0.25 mM EDTA, 0.01% NP-40, 1 mM MgCl2, 8% glycerol, 100 μg/ml BSA, 1 mg/ml Poly(dI-dC)], then loaded on a 6% acrylamide gel and run in standard 1× TBE, 1% glycerol. The gel was blotted to a positively charged Nylon membrane (Roche, Mannheim, Germany) in a Mini-PROTEAN Tetra Cell (Bio-Rad) in Tris glycin buffer. Protein–DNA complexes were fixed to the membrane by UV crosslinking. The DIG label was detected by DIG luminescent detection (Roche, Mannheim, Germany).

### Normalization of Repression Activity to Protein Concentration

TBX3 and its mutant derivatives differed in concentration in transfected cells, even though they were expressed from the same type of vector. This departure from uniform expression contributes to differences in repressive activity between the TBX3 variants. Repression was calculated from FLuc activity values by normalizing these values to the control (=100%). These values were subtracted from 100, yielding 0 repression for the control, and values between 0 and 100 for TBX3 and its derivatives. The dose response curves of p21-Luc repression by TBX3 and TBX3dm were approximately linear in the range between 8.3 and 25 ng with about 0.5% change in repression per nanogram of expression vector. Using the measured relative protein levels (Western blot), we extrapolated repression by the mutant proteins to the level observed for normal TBX3.

### Cell Culture and Transfection

COS-7 cells were cultured in Dulbecco’s Modified Eagle’s Medium (DMEM) (Biochrom AG, Berlin; Germany) containing 10% fetal calf serum (FCS, Biochrom AG) and 1% penicillin (10,000 U/ml)/streptomycin (10,000 μg/ml) (PS, Biochrom AG). Twenty-four hours before transfection 10^5^ cell/ml were cultured in DMEM/FCS in the absence of antibiotics and grown at 37°C, 5% CO_2_ to 70–90% confluence. Transient transfection was performed with 3 μl/ml FuGENE^®^ HD Transfection Reagent (Promega, Mannheim, Germany). In cycloheximide inhibition experiments, cycloheximide (Sigma-Aldrich, Taufkirchen, Germany) was added to 25 μg/ml.

### Measurement of Firefly Luciferase Activity

In the 96-well format, cells (100 μl) were transfected with 25 ng of reporter vector and 25 ng of TBX expression vector. Forty-eight hours after transfection, the supernatant was removed, cells were washed with PBS and then lysed for 30 min at room temperature in Passive Lysis Buffer (Promega, Mannheim, Germany). Twenty microliters of lysate were measured after addition of 40 μl luciferase agent (Luciferase Assay System, Promega) in white 96-well microplates (Nunc/Thermo Scientific, 136101, Schwerte, Germany) in a GloMax^®^ 96 Microplate Luminometer (Promega). Premeasurement delay was 2 s, the measurement took 10 s.

### Western Blot Analysis

Bacterial cells or partly purified protein fractions were supplied with 6× Laemmli sample buffer to a final concentration of 1× sample buffer (63). COS-7 cells were transfected for 48 h (12-well plates) with TBX expression constructs, trypsinized, and resuspended in PBS. After pelleting (5 min, 13,000 rpm in an Eppendorf microcentrifuge) cells were resuspended in 25–50 μl urea lysis buffer (7 M urea, 2 M thiourea, 5 mM DTT, 2% Chaps, 0.01% Proteinase Inhibitor Cocktail (Sigma-Aldrich P8340), 1 mM PMSF). Cells were lysed for 30 min on ice. Protein concentration was determined with a Bradford assay (Roti-Quant, Roth, Karlsruhe, Germany). Appropriate volumes of cell lysate were combined with 6× Laemmli sample buffer and loaded on an 8 or 10% SDS gel. PageRuler™ Prestained Protein Ladder (Thermo Scientific) was used to calibrate gel mobilities. Blotting to a PVDF membrane (Amersham Hybond™-P, GE Healthcare, München, Germany) and detection procedure was as described above in the EMSA protocol. FLAG epitope was detected with mouse monoclonal M2 (1:1000–2000), alpha-tubulin with mouse monclonal B5-1-2 (1:1000) (both Sigma-Aldrich). HRP-tagged goat anti-mouse IgG was used as a secondary antibody (1:10,000, Jackson ImmunoResearch, Dianova, Hamburg, Germany).

For pixel density analysis of fluorographs, several exposures were taken and analyzed using the ImageJ program^2^.

### RNA Isolation and Quantitative PCR

RNA was isolated 48 h after transient transfection using RNeasy kit (QIAGEN, Hilden, Germany) following the company’s instructions. cDNA was synthesized using Superscript III First-Strand Synthesis System for RT-PCR (Invitrogen) and oligo-dT primers according to instructions. qPCR reactions were performed in MicroAmp^®^ Fast Optical 96-Well Reaction Plates with Barcode (Life Technologies, 4346906) in a StepOnePlus™ Real Time PCR-System (Applied Biosystems, Darmstadt). Two hundred nanograms of cDNA, 0.1 μl each of forward and reverse primers (20 μM), and 10 μl of SYBR Green mix (KAPA SYBR FAST ABI Prism, Peqlab, Erlangen, Germany) were combined in a final volume of 20 μl. Cycling conditions were 3 s 95°C, 30 s 60°C followed by a melting curve analysis of the PCR product. Data were analyzed with the StepOnePlus™ software. Data were normalized to beta-actin (primers 1760/1761). *TBX3* expression was measured with primers 1924/1925, *p21* expression with primers 1772/1773 (see Table S5 in Supplementary Material).

### Statistical Analysis

Statistical analysis of cell culture experimental data was performed with GraphPad Prism (Version 5). Inflated *p*-values due to multiple comparisons between groups were adjusted by the Bonferroni–Holm procedure (64). The following significance levels were used: *<0.05, **<0.01, and ***<0.001. Significance assessment of *TBX* mutation type distributions in the ICGC database was done by calculating the respective probability for observing the actual or a more extreme distribution of the mutations between the different TBX3 domains. For this, an urn model was used with the number of mutations representing the number of draws. Since mutations can repeatedly occur at the same locus (“drawing with replacement”), a binomial distribution with the ratio of domain length to total TBX3 length as expectation value under the null hypothesis was chosen. The resulting *p*-values were doubled to correspond to two-tailed tests. Calculations were performed with SAS^®^ for Windows^®^ version 9.4 (SAS Institute Inc., Cary, NC, USA).

## Results

### Degree of Conservation of Mutated Amino Acids in TBX3

**Figure 1:**
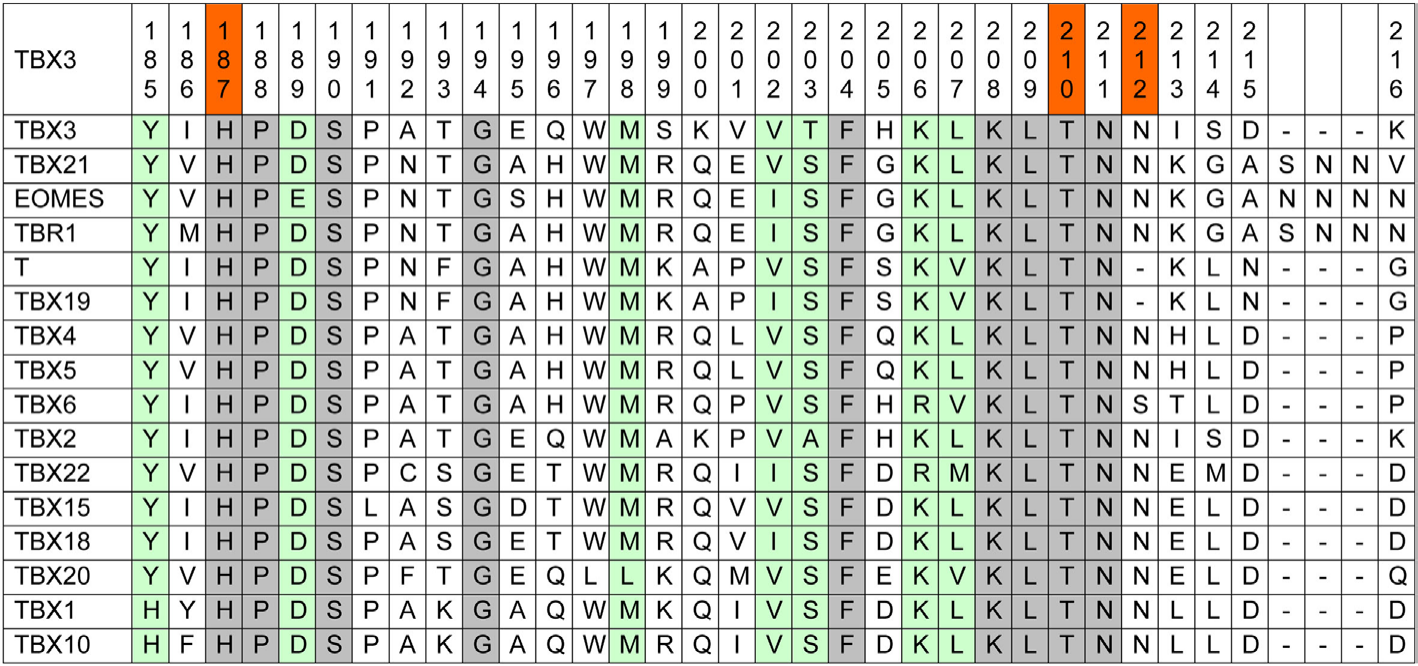
**Human TBX proteins in alignment with TBX3**. About one-sixth of the T-box, containing the mutations under consideration, is shown (TBX3 from 185 to 216). The complete TBX3 T-box encompasses residues 104–288 (66). Fully conserved amino acids are marked gray and T-box positions containing similar amino acids are green. In the top line, the three investigated mutations are marked red.

Amino acid conservation is used as one parameter to assess whether a mutation is likely to impart driver properties to the affected protein (65). Stephens and colleagues observed full conservation of the TBX3 residues, which were mutated in their collection of breast cancer sequences among orthologous animal Tbx3 proteins (1). Because of the strong phylogenetic conservation of the T-domain, we used a more stringent alignment containing all paralogous human TBX proteins. This alignment (Figure 1) shows that the mutation p.T210delT affects a fully conserved residue.

p.N212delN, which was identified in three cases of breast cancer (1, 61), affects a position that is largely conserved, only TBX6 showing a deviating serine. However, in Brachyury (T) and in TBX19, this residue is lacking, suggesting that it is not essential for Tbx function.

The mutation p.H187Y, a potential driver mutation identified in two cases of breast cancer, affects a fully conserved histidine (61).

Kandoth et al., in their analysis of 12 different cancer types, identified 33 further missense mutations in TBX3, 12 of which (36%) map to the T-domain [as defined by Porsch et al. (66)]. The T-domain makes up 27% of the TBX3 protein (743 amino acids), indicating only a slight enrichment of missense mutations in this part of the protein. In addition to p.H187Y, two further breast cancer mutations map to the TBX3 T-domain in the data set analyzed by Kandoth et al.: p.L112F and p.W113R. Both residues are fully conserved among human TBX proteins.

In addition to missense mutations, Stephens and colleagues described three frameshift mutations in TBX3 whose breakpoints cluster within 30 amino acids of the T-domain (1). As an example of a truncated protein, we also analyzed p.Y163fs2*.

### DNA-Binding Properties of Mutant Proteins

**Figure 2:**
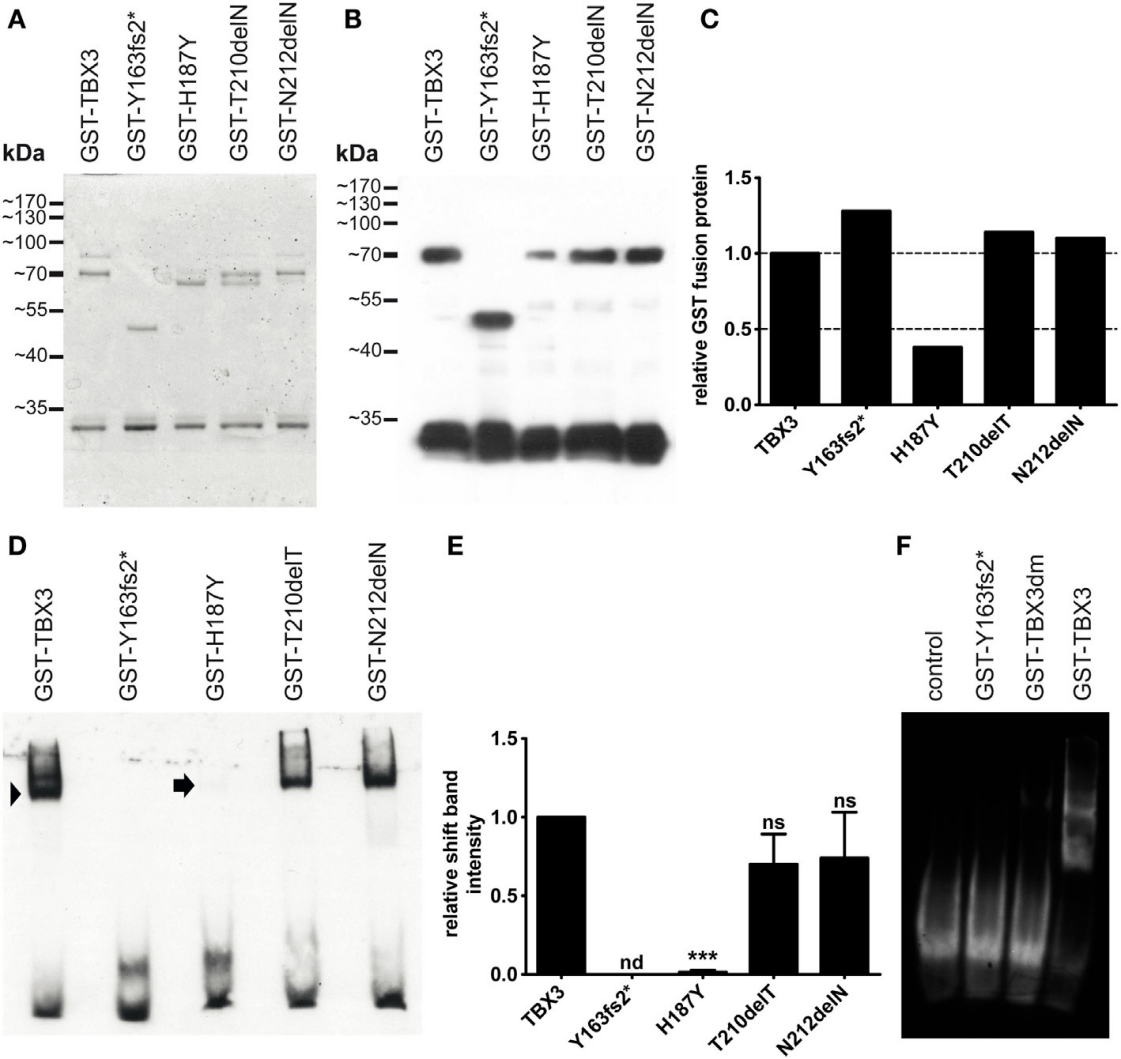
**Bacterial expression and DNA binding of mutant TBX3 proteins**. **(A)** GST-TBX3 protein variants after purification by glutathione affinity chromatography. Equal amounts of peak eluate protein were separated on a 10% SDS gel and stained with Coomassie Blue. Normal and point mutant protein fractions yielded a full-length band of 70 kDa [calculated molecular weight of the GST-TBX3(1–342) fusion protein is 65.4 kDa] and a band with ~30 kDa mobility. GST-TBX3(fs163fs2*) migrated with the expected mobility of ~48 kDa. **(B)** Anti-GST Western blot. A gel as shown in **(A)** was blotted and probed with anti-GST. Both the 70 and the 30-kDa band reacted with the antibody. Additional bands visible in the Coomassie-stained gel between 65 and 80 kDa were not immunoreactive. **(C)** Quantification of fusion band expression of the blot shown in **(B)**. **(D)** Electrophoretic mobility shift assay of GST-TBX3 protein variants. Equal amounts of fusion protein as determined in **(C)** were employed. The GST-TBX3(1–342) wildtypic fusion protein effectively shifted the consensus T-box binding element oligonucleotide (arrowhead), the point mutant derivatives T210delT and N212delN were slightly less efficient, H187Y yielded only a faint signal (arrow). **(E)** Quantification of shift-band intensity. The amount of oligonucleotide shifted by TBX3 was set to 1 (*n* = 4). (n = not detected, ns = not significant) **(F)** EMSA comparison of p.Y163fs2* and the negative control mutant TBX3dm. Control is shift oligonucleotide alone.

We tested DNA binding of the bacterially expressed mutant proteins by EMSA. For all variants, we used an N-terminal fragment (1–342) in order to reduce degradation to which the full-length protein is susceptible. The fragment was derived from the long TBX3 + 2a splice form (67). The 1–342 fragment extends C-terminally for some 50 amino acids beyond the T-domain. The protein length of 342 was chosen, because the paralogous proteins TBX2 and TBX3 start to diverge more strongly beyond this position. All fragments contained an N-terminal GST tag (glutathione S-transferase) for affinity purification. Mutations were introduced by site specific mutagenesis. The mutant proteins were expressed in *E. coli* and subsequently purified by glutathione affinity chromatography. Figure 2A shows that all proteins were obtained with similar purity. A Western blot of SDS gel-separated proteins (Figure 2B) shows that the fast band at around 30 kDa, which is present in all preparations, was also immunoreactive to anti-GST. The molecular mass of GST is 26.7 kDa, indicating that GST is partly cleaved from TBX3 very close to the TBX3 N-terminus, possibly in the protease cleavage site, which is present in pGEX-expressed proteins at the junction between GST and cargo. The intensity of the GST-TBX3 fusion bands was measured (Figure 2C) so that equivalent amounts of normal and mutant GST-TBX3 fragments could be tested by EMSA for binding to a palindromic Tbx consensus oligonucleotide (68). The normal protein produced a single strong shift band. The binding affinities of p.N212delN and p.T210delT were slightly reduced compared to wild type (Figure 2D). The reduced affinity of p.T210delT and p.N212delN was not statistically significant (Figure 2E), even though we observed the same pattern of attenuated binding in all EMSA gels. Binding of p.H187Y was only obvious when the gel was overexposed. No DNA binding was observed with p.Y163fs2* (Figures 2D–F) and p.G129A/R130S (TBX3dm, engineered as a binding-deficient mutant, see below) (Figure 2F) under these conditions.

### Reporter Gene Repression in Transfected Cells: The Role of DNA Binding

TBX3 was first implicated in tumorigenesis because it was found to suppress cellular senescence, could immortalize primary embryonic fibroblasts, and could transform this cell type in combination with Myc or activated Ras (13, 16, 35). In the suppression of cellular senescence, transcriptional repression of *cyclin-dependent kinase inhibitor 1A* (*p21*) and *2A* (in human: *p14ARF*; in mouse: *p19ARF*) has a key role. The repression by TBX3 is direct and repression activity of TBX2/3 variants can be tested on *p21* promoter constructs in transient transfection assays (32, 69). Since the presumptive TBX3 driver mutations affected DNA binding, we first tested whether p21-Luc could still be repressed by a DNA-binding-deficient mutant. We used the TBX3 double mutant p.G129A, R130S (TBX3dm). Single mutations in homologous position have previously been shown to cause loss of DNA binding in other Tbx proteins [TBX5: G80R (70), Omb: R355K (71), see also TBX2 (R122E, R123E) (72)].

**Figure 3:**
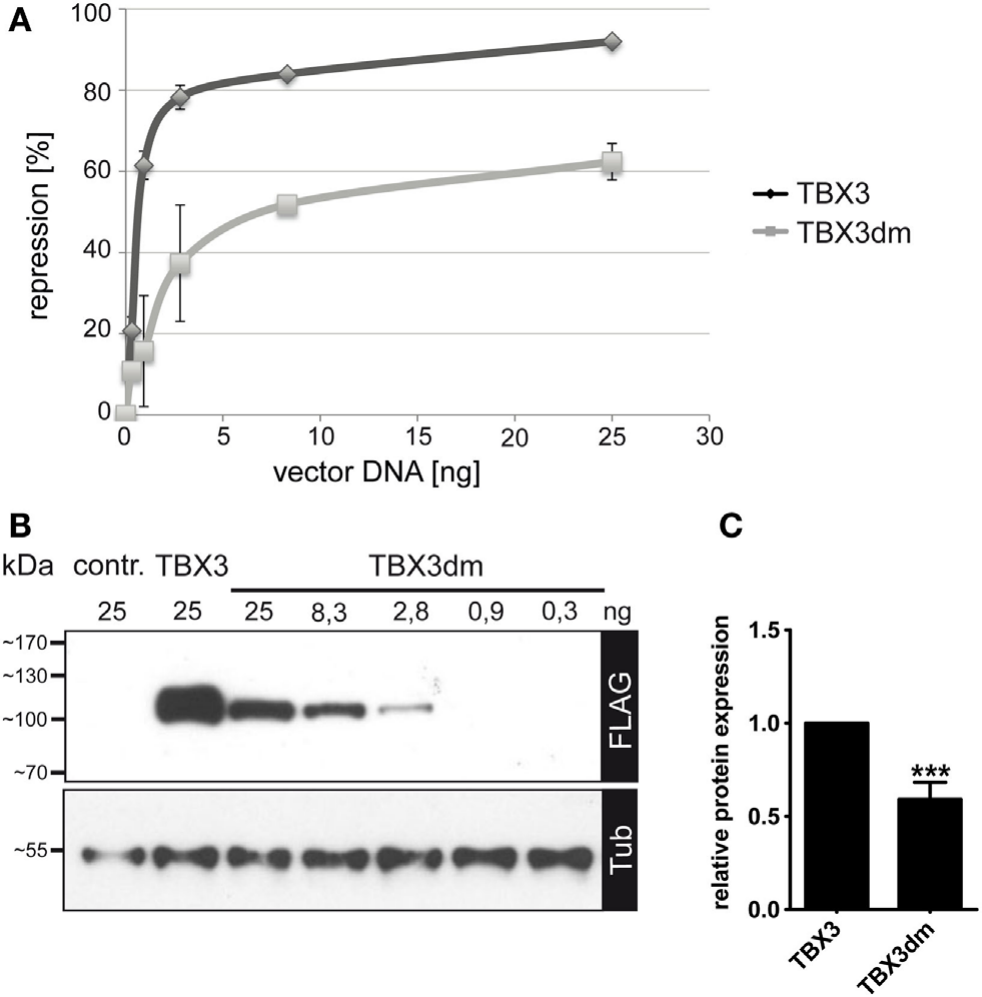
**Repression of p21-Luc reporter by TBX3 and the DNA binding-deficient mutant TBX3dm**. **(A)** Comparison of p21-Luc repression by TBX3 and TBX3dm in transfected COS-7 cells. In all assays, a total of 25 ng of the expression vector was used, with a varying ratio of empty to TBX vector. In the control, 25 ng of empty expression vector was transfected. Repression of the p21-Luc reporter was measured at six different concentrations of TBX3 or TBX3dm. Luc activity in the absence of TBX3 was defined as 0 repression (*n* = 3). **(B)** Comparison of TBX3 and TBX3dm expression. TBX3dm expression is shown in a dilution series of its expression vector. Protein expression was visualized by Western blot of cell extract and FLAG immunodetection. Protein load was controlled with anti-alpha-tubulin. **(C)** Relative expression of TBX3 and TBX3dm. COS-7 cells were transfected with 25 ng expression vector (*n* = 6).

In COS-7 cells, the p21-Luc promoter construct was repressed by TBX3 in a dose-dependent fashion. Normal TBX3 caused 80–90% repression of reporter activity at our highest concentration of TBX vector (25 ng/assay). TBX3dm generally reached 60% repression (Figure 3A). Both proteins at 25 ng approached saturation. The dose-dependence curves show that TBX3 reached 60% repression already at a concentration of <1 ng/assay, while TBX3dm required 25 ng/assay to reach this degree of repression. The data indicate that part of the repression caused by TBX3 does not require DNA binding. The mutant p.Y163fs2*, which lacks both an intact DNA-binding domain and the entire C-terminal domain, failed to repress the p21 reporter (Figure S1 in Supplementary Material).

**Figure 4:**
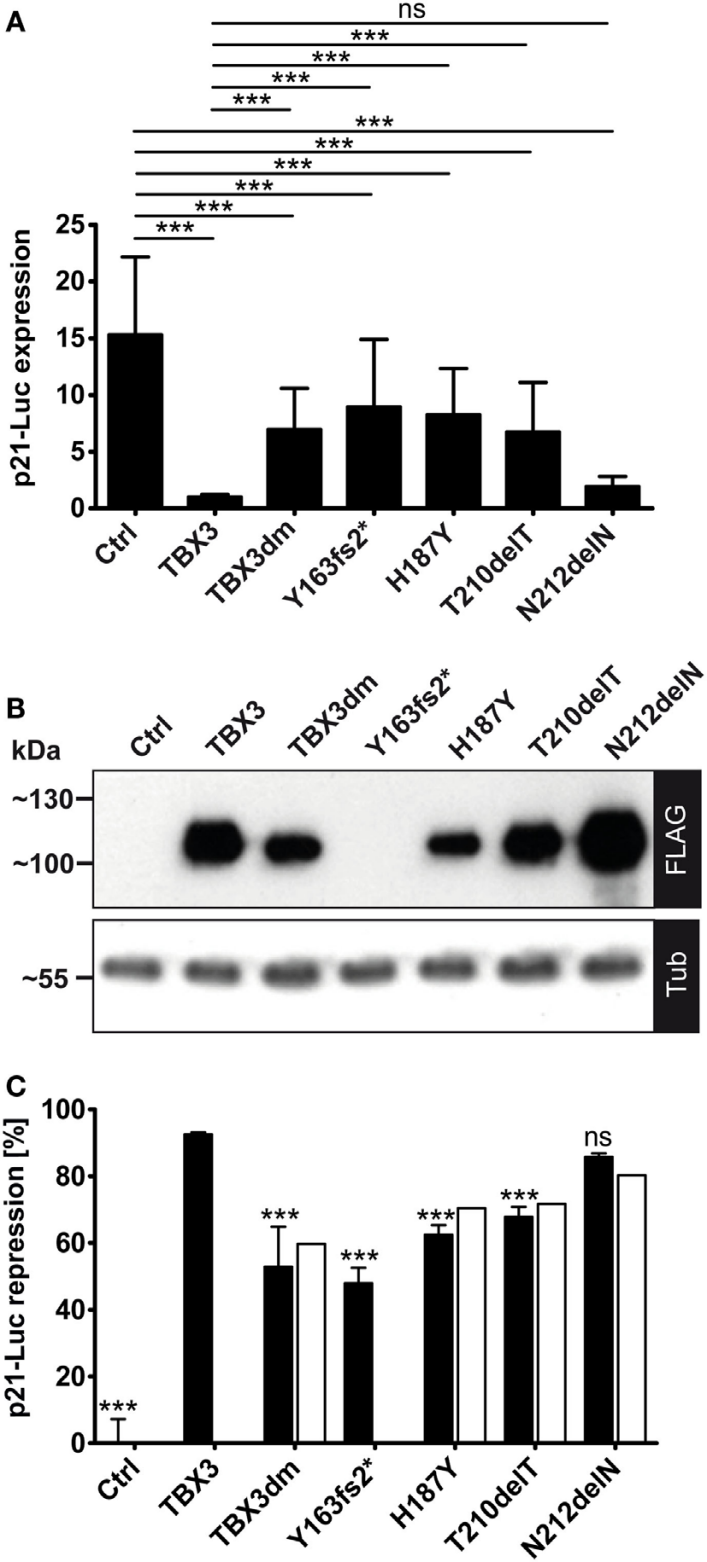
**Repression of a p21-Luc reporter by TBX3 and mutant derivatives**. **(A)** The repression of the p21-Luc reporter was measured with 25 ng of expression vector encoding the normal or mutant TBX3 proteins. For control, empty expression vector was transfected. Luc expression is in arbitrary units. The Luc data from six independent experiments were normalized to TBX3 (=1). Average and SD are shown. TBX3dm, Y163fs2*, H187Y, and T210delT were significantly weaker repressors than normal TBX3 but also differed significantly from control. The mutant N212delN did not differ significantly from normal TBX3; hence, was an effective repressor. **(B)** Quantification of protein expression. Protein expression was visualized by Western blot of cell extract and FLAG immunodetection. Protein load was controlled with anti-alpha-tubulin. **(C)** Normalization of Luc repression by TBX3 and mutant derivatives to their respective protein concentration. The luciferase activity values of one experiment, for which the protein expression data are shown in **(B)**, were normalized to the control (=100%). To obtain repression data, these values were subtracted from 100 (0% repression in the control) and shown as black columns (error bars are SD, *n* = 6 technical replicates). The values after normalization to the different protein levels (see Materials and Methods) are presented by white columns.

TBX3dm protein abundance in transfected cells reproducibly was less than that of normal TBX3 (as determined by anti-FLAG immunoblotting: 0.59 ± 0.08, *n* = 6) (Figures 3B,C and 4B). The repression activity of TBX3dm at 25 ng (60% repression) should, thus, be compared to TBX3 at 15 ng (85% repression) (Figure 3A). Because of the shallow slope in this part of the dose–response curve, TBX3 remains a more effective inhibitor than TBX3dm even when adjusted for differences in protein abundance.

### Reporter Gene Repression in Transfected Cells: The Effect of Presumptive Driver Mutations

We used the p21-Luciferase assay to determine how TBX3 activity was affected by the putative driver mutations. Full-length proteins, C-terminally tagged with a FLAG tag were used, with the exception of p.Y163fs2* where the untagged truncated protein was tested. As observed above (Figure 3A), the DNA-binding-defective double mutant TBX3dm repressed p21-Luc 1.5- to 2-fold. A similar repression was observed for the other binding-deficient mutant p.Y163fs2*. Surprisingly, the mutants that were less impaired in DNA binding, p.H187Y and p.T210delT, did not differ significantly from TBX3dm. However, the repression activity of p.N212delN was close to that of TBX3 (Figure 4A). While the differences in p21-Luc repression between TBXdm, Y163fs2*, H187Y, and T210delT were small, the same relative order was observed in all experiments.

We did not normalize the data in Figure 4A to the expression of a co-transfected vector because we noted that the expression of *Renilla* luciferase from the normalization vector pGL4.74 (pRL-TK, Promega) was also modulated by TBX3 and its mutants. This also holds for other normalization constructs (29). As noted above for TBX3dm, the expression level for mutant proteins differed in a characteristic way from normal TBX3. TBX3dm, H187Y, and T210delT were less abundant than TBX3 (~2×, ~3×, and ~1.5×, respectively), while N212delN was always more abundant (up to 1.5×) (Figure 4B). We normalized the repression to a uniform protein concentration (see Materials and Methods). This correction led to stronger apparent repression activity of the proteins expressed at a lower level than TBX3, and *vice versa*. Overall, the effect of normalization was small (Figure 4C). The normalization could not be performed for the untagged p.Y163fs2*.

### Repression of Endogenous *p21* by TBX3 and Mutant Derivatives

**Figure 5:**
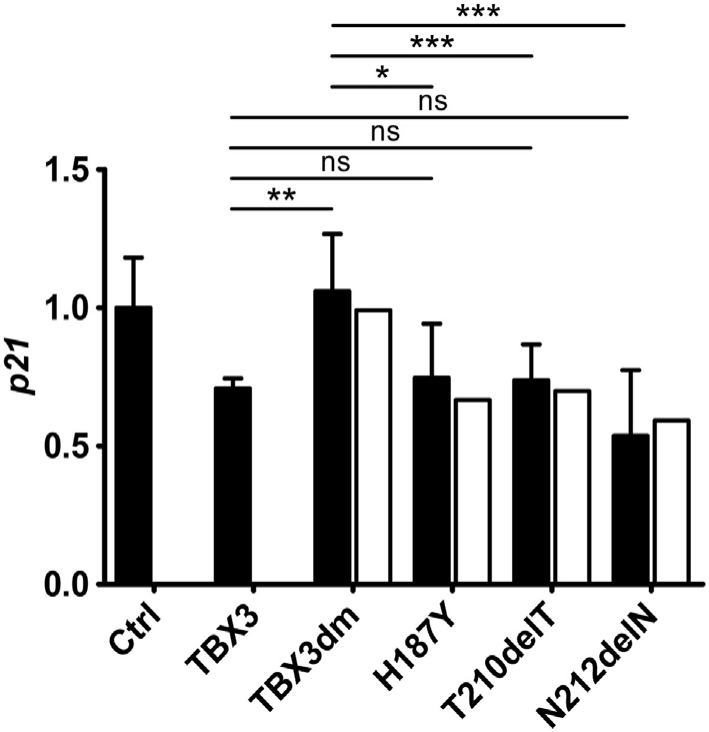
**Repression of the endogenous *p21* gene by TBX3 and mutant derivatives**. COS-7 cells were transfected with 50 ng of expression vector encoding TBX3 or its mutants (the empty vector was transfected in the control experiment). RNA was isolated and the level of p21 transcripts was determined by real-time qPCR. The p21 level is normalized to the control (1.0, black columns, error bars are SDs, *n* = 3 with three technical replicates each). To correct for differences in the amount of TBX protein, an adjustment was performed as described in Section “Materials and Methods” (white columns). Only TBX3dm differed significantly in *p21* repression from normal TBX3, the three single mutants did not.

Transiently transfected reporter constructs cannot be expected to render the regulatory complexity of the endogenous gene. We, therefore, determined by real time qPCR how TBX3 and its mutant derivatives affected endogenous *p21* transcription. TBX3 repressed endogenous *p21* although to a lesser degree than the p21-Luc reporter (Figure 5). Of the four tested mutants, only the DNA-binding-deficient TBX3dm did not repress the endogenous gene. The three putative driver mutant proteins containing point mutations were as repressive on endogenous *p21* as normal TBX3 (the untagged frame shift mutant p.Y163fs2* was not tested). This still held when the *p21* expression values were normalized to protein abundance. The repressive effect of the TBX3 mutants, thus, differed between endogenous *p21* and the transfected p21-Luc reporter. Mutant TBX3 proteins with residual DNA-binding affinity (H187Y, T210delT, and N212delN) effectively repressed the endogenous *p21* gene but TBX3dm failed to do so. However, on transfected p21-Luc, TBX3dm repressed transcription about twofold, similar to the other strongly DNA-binding-defective mutant proteins H187Y and T210delT (Figure 4C).

### Differential Expression of Mutant TBX3 Proteins

The expression level of the five FLAG-tagged TBX3 variant proteins, as determined by Western blotting, differed in a characteristic manner (Figure 4B). This pattern was observed in five independent transfection experiments and, thus, was unlikely to be produced by stochastic fluctuations.

**Figure 6:**
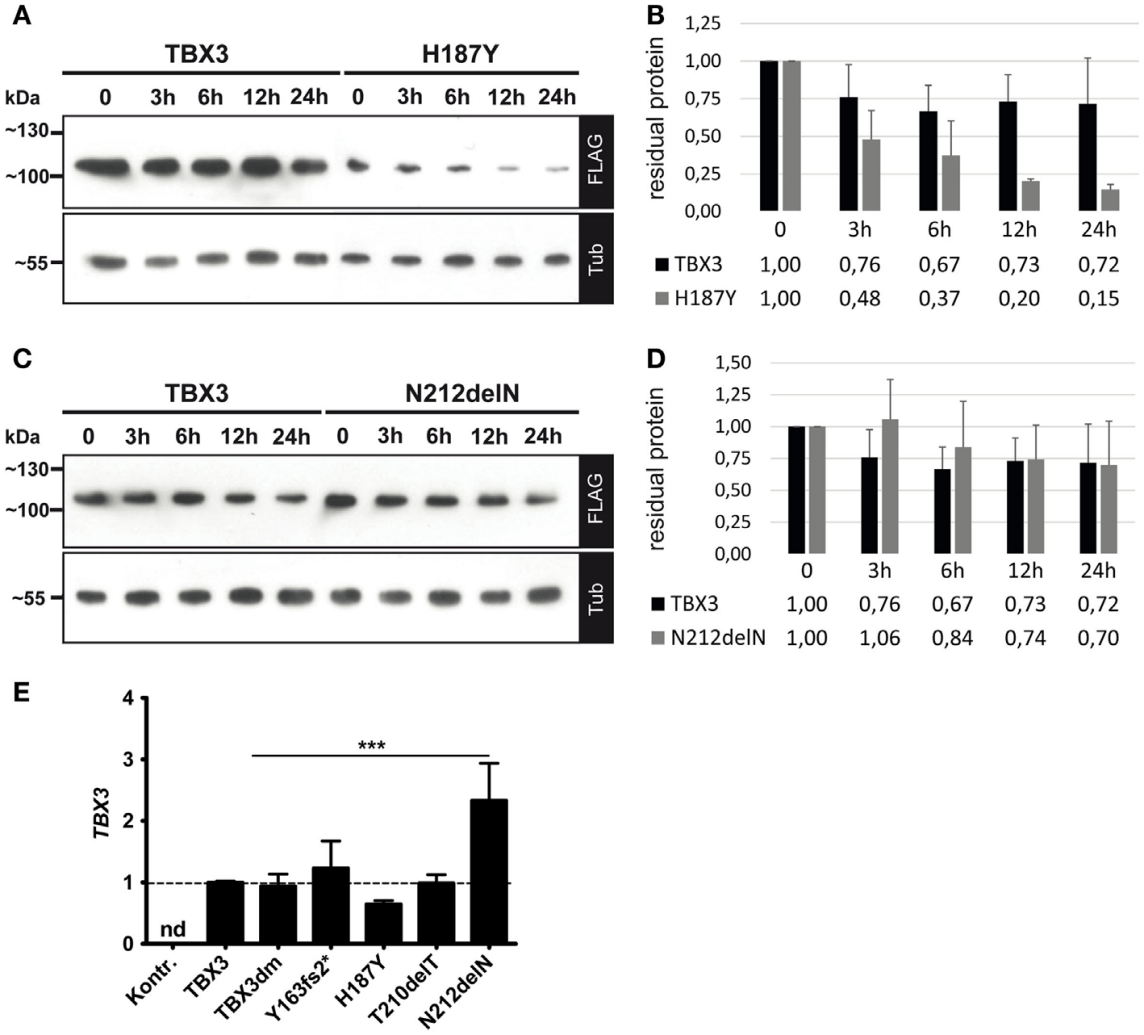
**Protein stability and transcript abundance of TBX3 and mutant derivatives**. COS-7 cells were transfected with 50 ng of expression vector encoding TBX3 or its mutants. Twenty-four hours after transfection cycloheximide (CHX) was added to 25 μg/ml. Cells were lysed at the indicated time points. Protein level was quantified by Western blot of cell extract and FLAG bioluminescence immunodetection. Protein load was controlled with anti-alpha-tubulin. **(A,B)** TBX3 and H187Y, **(C,D)** TBX3 and N212delN. **(E)** Quantification of TBX3 transcript levels by real time qPCR. COS-7 cells were transfected with 25 ng of expression vector encoding TBX3 or its mutants (the empty vector was transfected in the control experiment). RNA was isolated 48 h after transfection and analyzed. The data are normalized to TBX3. No TBX3 transcription was detected (nd) in cells transfected with empty vector. The higher level of N212delN RNA relative to normal TBX3 was statistically significant (*n* = 3).

All TBX3 variants were expressed from the same expression vector. The different protein accumulation which we observed, therefore, could be due to differential protein stability. We determined protein stability of TBX3, H187Y, and N212delN by blocking protein synthesis with cycloheximide (73) and following protein abundance over 12–24 h. Protein levels were normalized to alpha-tubulin, which appeared stable over this period. The TBX3 level declined to 0.73 within 12 h (SD = 0.18, *n* = 6), whereas H187Y declined to 0.2 (Figures 6A,B), i.e., at least threefold faster than the normal protein. N212delN before addition of cycoheximide was slightly more abundant than TBX3 and was similarly stable (Figures 6C,D); N212delN declined to 0.74 within 12 h (SD = 0.26, *n* = 3). Thus, differential protein stability did not account for the higher steady state abundance of N212delN. Therefore, we also determined the transcript level of normal and mutant TBX3 in transfected cells by real-time PCR. Surprisingly, transcript abundance of H187Y was slightly lower and that of N212delN significantly higher than that of TBX3, indicating that difference in RNA production or turnover contributes to the differences in protein levels (Figure 6E).

### Statistical Considerations of Somatic *TBX3* Mutations in Cancer Tissues

The somatic mutation database of the International Cancer Genome Consortium (ICGC)^3^ lists 149 somatic mutations in *TBX3*. In order to draw inferences about the relevance of these mutations, we used the mutations in the other human *TBX* genes as reference. Table S1 in Supplementary Material shows the distribution of somatic mutations in the 16 human *TBX* genes in different cancer tissues. The breast cancer data (BRCA-US) derive from TCGA project in which somatic mutations from about thousand donor tissues were analyzed (61, 74). It is obvious that in breast cancer, *TBX3* has the highest number of mutations of all *TBX* genes.

In Table 1, these numbers are normalized to the total number of mutations in a given *TBX* gene across the different cancer types. This normalization allows the comparison between different *TBX* genes. In breast cancer, *TBX3* is the most frequently mutated of all *TBX* genes (17.6%). The mutation rate averaged over all 16 *TBX* genes is 3.6% corresponding to a 4.7-fold enrichment for *TBX3*.

**Table 1:** **Relative frequency of *TBX* mutations in ICGC somatic cancer mutation data base**.

	EOM ES	T	TBR 1	TBX 1	TBX 2	TBX 3	TBX 4	TBX 5	TBX 6	TBX 10	TBX 15	TBX 18	TBX 19	TBX 20	TBX 21	TBX 22	Average	Total
Liver cancer – RIKEN, JP	8.6	9.2	10.5	0	5.9	7.2	9.9	13.3	2.4	5.2	17.1	16	16	22.8	11.7	14.7	10.66	396
Esophageal adenocarcinoma – UK	6.5	9.2	9.5	9.7	1	3.9	10.5	16.4	1.2	2.1	16.1	14	10.7	16.3	9.6	0	8.54	335
Pancreatic cancer – AU	12.9	8.4	9.5	11.8	7.8	11.1	11.7	13.1	6	0	0	5.9	9.3	12.6	12.8	12.3	9.08	255
Skin cutaneous melanoma – TCGA, US	12.9	10.7	14.3	6.5	7.8	5.9	7	3.8	13.3	18.8	8.9	4.8	10	8.5	8.5	6.7	9.28	239
Ovarian cancer – AU	4.3	4.6	1	12.9	2.9	6.5	6.4	8.7	3.6	8.3	12.9	9.4	5.3	6.8	3.2	8.6	6.59	235
Gastric adenocarcinoma – TCGA, US	7.5	11.5	11.4	5.4	9.8	5.2	4.7	4.9	19.3	10.4	0.7	4.4	6.7	1	6.4	4.9	7.14	160
Liver cancer – FR	1.1	1.5	1.9	2.2	2.9	0.7	4.7	2.3	7.2	3.1	5.1	14.7	5.3	0.7	6.4	2.5	3.89	146
Colon adenocarcinoma – TCGA, US	8.6	7.6	7.6	6.5	5.9	5.2	7	3.3	13.3	7.3	2.6	3.1	3.3	3.1	4.3	3.1	5.74	137
Liver cancer – NCC, JP	7.5	2.3	6.7	9.7	3.9	2.6	4.1	5.6	3.6	2.1	2.3	3.1	4.7	4.1	5.3	4.9	4.53	124
Renal cell cancer – EU/FR	1.1	1.5	2.9	5.4	8.8	1.3	8.8	4.1	1.2	2.1	4.4	5.9	3.3	3.4	5.3	1.2	3.79	124
Lung squamous cell carcinoma – TCGA, US	4.3	6.9	4.8	0	5.9	7.8	0.6	2.3	2.4	4.2	0.5	3.5	3.3	2.7	5.3	8	3.91	101
Pancreatic cancer – CA	3.2	3.1	1.9	3.2	3.9	3.9	1.8	4.1	1.2	1	4	1.8	9.3	2	4.3	1.8	3.16	95
Breast cancer – TCGA, US	3.2	3.1	3.8	2.2	2.9	17.6	2.3	2.1	1.2	5.2	0.9	1.5	2.7	1.4	2.1	6.1	3.64	92
Oral cancer – IN	2.2	0	1	0	0	0	0.6	0.3	0	8.3	13.3	0	0	0.3	0	0	1.63	71
Breast triple negative/lobular cancer – UK	0	0.8	0	9.7	1	2.6	3.5	1.8	1.2	1	2.1	2.2	3.3	3.1	1.1	0.6	2.13	65
Malignant lymphoma – DE	1.1	1.5	1	1.1	1	4.6	2.3	2.8	2.4	1	1.6	1.3	1.3	1	0	6.7	1.92	60
Lung cancer – KR	0	4.6	1	2.2	6.9	0.7	0	2.1	3.6	1	1.6	0.9	1.3	2	0	5.5	2.09	57
Thyroid cancer – SA	1.1	1.5	3.8	4.3	7.8	1.3	1.8	1.3	6	4.2	0.7	0.4	0	0.3	3.2	1.8	2.47	50
Pancreatic cancer endocrine neoplasms – AU	0	0.8	0	1.1	1	1.3	1.8	1	1.2	0	1.2	1.8	0	2.4	0	1.2	0.93	35
Bladder urothelial cancer – TCGA, US	2.2	3.1	1.9	0	2.9	2.6	1.8	1.8	2.4	1	0.5	0	0.7	0	0	0.6	1.34	32
Pediatric brain cancer – DE	0	0	1	0	0	0	1.2	1	0	0	1.2	2.2	1.3	0.7	0	1.8	0.65	29
Acute myeloid leukemia – KR	0	2.3	0	2.2	2	0	1.8	0	0	5.2	0.2	0.2	0	0	5.3	0	1.20	22
Rectum adenocarcinoma – TCGA, US	0	0.8	1.9	1.1	3.9	1.3	1.2	0	0	2.1	0.2	0.2	0	0.7	1.1	1.8	1.02	22
Brain glioblastoma multiforme – TCGA, US	1.1	1.5	1	1.1	0	0	0	1	1.2	0	0.2	0.4	0	0.3	1.1	1.8	0.67	18
Prostate adenocarcinoma – TCGA, US	2.2	0	0	0	0	2.6	0	0.3	0	0	0	0.9	0.7	1.4	0	0	0.51	16
Brain lower grade glioma – TCGA, US	0	0.8	0	1.1	2	0	0	0.5	0	2.1	0.2	0.2	0	0.3	0	0.6	0.49	12
Kidney renal clear cell carcinoma – TCGA, US	2.2	0	1	0	0	0	1.2	0	3.6	0	0	0.2	1.3	0	1.1	0	0.66	12
Bladder cancer – CN	1.1	0.8	0	0	0	0.7	1.8	0.5	0	2.1	0	0	0	0.3	0	0	0.46	11
Prostate adenocarcinoma – CA	1.1	0	0	0	0	0	0	0.8	0	0	0.9	0	0	0.3	0	0	0.19	9
Kidney renal pap. cell carcinoma – TCGA, US	1.1	0	0	0	2	1.3	0.6	0	1.2	1	0	0	0	0	0	0	0.45	8
Prostate adenocarcinoma – UK	1.1	0.8	0	0	0	1.3	0.6	0	0	0	0	0.4	0	0	0	0.6	0.30	8
Early onset prostate cancer – DE	0	0.8	0	0	0	0	0.6	0	0	0	0.5	0.2	0	0	0	0.6	0.17	6
Ovarian serous cystadenoca – TCGA, US	2.2	0	1	1.1	0	0	0	0.3	1.2	0	0	0	0	0	0	0	0.36	6
Head and neck thyroid carcinoma – TCGA, US	0	0	0	0	0	0	0	0.3	0	1	0	0.4	0	0.3	0	0	0.13	5
Esophageal cancer – CN	0	0	0	0	0	0.7	0	0.3	0	0	0	0	0	0.7	0	0	0.11	4
Gastric cancer – CN	0	0.8	0	0	0	0	0	0	0	0	0	0	0	0.3	0	0.6	0.11	3
Benign liver tumor – FR	0	0	0	0	0	0	0	0	0	0	0	0	0	0	2.1	0	0.13	2
Chronic lymphocyclic leukemia – ES	0	0	0	0	0	0	0	0	0	0	0	0	0	0	0	0.6	0.04	1
Total	100	100	100	100	100	100	100	100	100	100	100	100	100	100	100	100	100	3003
																		

*The numbers of TBX mutations in 38 different somatic cancer genome projects (cf. Table S1 in Supplementary Material for absolute numbers) were normalized for each of the 16 human TBX genes. TBX3 mutations and data from the TCGA breast cancer project are highlighted by gray shading. The total number of TBX mutations identified in different cancer projects is given in the rightmost column. These numbers vary largely due to the number of tissues analyzed and due to cancer-intrinsic differences in mutation rate. In the TCGA breast cancer data set, TBX3 is the most frequently mutated TBX gene*.

In Table S2 in Supplementary Material, the mutation numbers are normalized to the number of mutations in the entire set of *TBX* genes. This serves to normalize mutations in a given gene to the average mutation frequency in a particular cancer tissue and to the different number of tissue samples analyzed. Breast cancer was conspicuous because 29.3% of all *TBX* mutations in this tissue were found in *TBX3*. This is not due to *TBX3* being a larger mutational target. *TBX3* (13.9 kb) is twofold smaller than the average of *TBX* genes (29.9 kb). Table 1 and Table S2 in Supplementary Material are sorted by the number of *TBX* mutations identified in a given cancer project. Results at the top of these tables are more significant than those at the bottom.

Human *TBX* gene size varies by more than 17-fold (between 6 and 106 kb), whereas variation in protein length is <2-fold. The ICGC data collection also contains intronic mutations such that mutation number per gene will be influenced by gene size. Table 2 gives the number of breast cancer mutations that affect the open reading frame (ORF) of *TBX* genes. Five types of mutations were considered: frameshift, missense, synonymous, stop codon gained, and in-frame deletion. Compared to the average occurrence of a particular mutation type in *TBX* genes, frameshift and in-frame deletion mutations in *TBX3* were strongly (12× and 16×) and missense mutations moderately (3×) enriched. This enrichment is characteristic for breast cancer. When the entire ICGC data set was analyzed for *TBX3* ORF mutations (ratio of complete ICGC to BRCA-US dataset ~12/1), 21 frameshift mutations were found, 15 of which arose in breast cancer (8.5-fold enrichment). This concentration of frameshift *TBX3* ORF mutations in breast cancer is highly non-random (*p* < 10^−7^). Similarly, two of five in-frame deletions occurred in the BRCA-US data set. The preponderance of these particular types of mutations in *TBX3* versus the other *TBX* genes points to an important role of TBX3 in breast cancer progression.

**Table 2:** **Mutations affecting the open reading frame of TBX genes in breast cancer**.

TBX gene	Protein length	Frame shift	Missense	Stop gained	In-frame del	Syn	Sum
EOMES	705		3				3
T	435	1	1			2	4
TBR1	682	1	2				3
TBX1	495		1			1	2
TBX2	712	1					1
TBX3	723	15	8	1	2	1	27
TBX4	545		2	1		1	4
TBX5	518	1	5			1	7
TBX6	436						0
TBX10	385	1		1		1	3
TBX15	602		4				4
TBX18	607		5			2	7
TBX19	448		1	1			2
TBX20	447		3				3
TBX21	535					2	2
TBX22	520		6	1		1	8
Sum		20	41	5	2	12	80
Average mut nr/TBX gene		1.25	2.56	0.31	0.12	0.75	

*The TCGA breast cancer data set was analyzed for five types of mutations affecting the open reading frame (ORF) of TBX genes. ORF length is in the second column. In the second but last column, synonymous codon changes (syn) are listed. The row at the bottom presents the mutation number averaged over 16 TBX genes. This allows to calculate the enrichment of particular mutation types in TBX3 (shaded)*.

**Figure 7:**
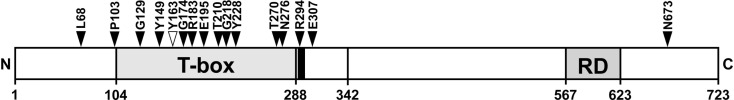
**Position of TBX3 frameshift mutations in breast cancer**. The position of 16 somatic frameshift mutations identified in primary breast cancer tumors is indicated on a schematic drawing of TBX3 [723 amino acids (aa)]. The positions of the T-box, the nuclear localization sequence [290–295 aa, black bar adjacent to T-box (35)], and the C-terminal repression domain (35) are shown as gray boxes. Fifteen of 16 mutations cluster in the N-terminal half of the protein, most of them in the T-box.

The position of breast cancer frameshift mutations in *TBX3* was highly non-random. Fourteen of 15 mutations lie in the N-terminal half of TBX3, 10 lie in the T-domain (Figure 7). Both distributions are highly unlikely to arise by chance (*p* < 10^−4^ and <2 × 10^−3^, respectively). The frameshift mutation, which we analyzed (Y163fs2*) and identified in a different cancer project (1), also fell into this pattern.

If breast cancer progression is associated with mutations that cause loss of DNA binding, this should also be reflected in TBX3 missense mutations. In the BRCA-US data set, eight independent missense mutations in *TBX3* were identified, H187Y occurring twice. Four of these lie in the T-domain [L112F, W113R, H187Y (2×)] (Table S3 in Supplementary Material). We expect that mutations of fully conserved residues of the T-domain are more likely to disturb DNA binding than mutations of non-conserved residues. L112F, W113R, and H187Y are fully conserved residues in all 16 human TBX proteins. We showed that DNA binding was severely compromised by H187Y (Figure 2C). A mutation affecting the tryptophane corresponding to TBX3 W113 was identified in TBX22 in a patient with X-linked cleft palate (W102C). The mutant TBX22 protein is inactive in DNA binding (75). In the BRCA-US data set, 13 missense mutations lie in the T-domain of *non-TBX3 TBX* genes. Of these, three affect fully conserved (23%), two functionally conserved (15%), and eight (62%) non-conserved residues. This closely corresponds to the distribution of conservation categories (25, 21, and 64%, respectively). The enrichment for conserved residues as targets of missense mutations in TBX3 supports the idea that loss of TBX3 T-box function is relevant for breast cancer progression. There was also a trend for conservation of the C-terminal TBX3 breast cancer mutations. About one-quarter of C-terminal residues are conserved between mammalian Tbx2 and Tbx3 proteins. Three of four C-terminal *TBX3* mutation affected such conserved amino acids.

## Discussion

### Protein Structural Considerations of the Investigated Putative Driver Mutations

Coll and colleagues have analyzed the structure of the TBX3 T-domain in complex with a 20-bp palindromic target sequence (76). In this structure, N212 is part of a loop that connects the short β-strands e and e′. The length of this loop varies in different Tbx proteins. As shown in Figure 1, the members of the Tbr1 subfamily (TBR1, TBX21, and EOMES) (77) contain an insertion of three amino acids in this loop. *Drosophila* Omb, too, the insect ortholog of Tbx3, contains such an insertion. Importantly, the e–e′ loop also contains the 20 amino acids insertion present in the splice variant TBX3 + 2a. TBX3 + 2a is the product on an alternative splice form, which contains the additional 60 bp exon 2a which is found in all mammalian *Tbx3* genes (69). Hoogaars and colleagues found no influence of the additional 20 amino acids on DNA-binding *in vitro*, on the interaction with Nkx2–5, and on the repressor function of Tbx3 on two natural promoters in transfected cells. Similarly, no difference was noted in the interaction with Sox4 (78). The issue is, however, controversial (79, 80). Our data show that shortening of the e–e′ loop had little effect on DNA-binding *in vitro* and target gene repression. While N212 is a strongly conserved residue among T-box proteins (mammalian members of the Tbx6 subfamily carry serine at this position, Figure 1), members of the T subfamily (Brachury and TBX19) lack the corresponding amino acid. This suggests that N212 is not essential for T-box function.

In the X-ray structure of the TBX3–DNA complex, T210 is part of the short β-strand e encompassing amino acids 208–211. These four amino acids are completely conserved in the T-box protein family (Figure 1). K208 and N211 interact with the target DNA both in TBX3 and in *Xenopus* Brachyury (76, 81). A structural change in this protein strand is, therefore, expected to have functional consequences. In TBX19, even a conservative mutation of the lysine, corresponding to K208 in TBX3, to arginine abolishes DNA binding and target gene activation (82). The mutation T209I in Tbx20 (T209 in Tbx20 corresponds to T210 in TBX3, see Figure 1) causes a reduction in the activation of an endogenous target gene in an *ex vivo* assay at low concentration of the mutant protein. However, at high concentration the mutant protein is as effective as normal Tbx20 (83). In this respect, Tbx20–T209I and TBX3–T210delT appear similar. T210delT, too, reduces but does not eliminate affinity for T-box target sequences.

H187 is completely conserved in the T-box protein family. In the TBX3/DNA structure, it lies in the β-strand C′, which is part of the seven-stranded β-barrel that forms the core of the T-domain. C′ does not contact the DNA but the H187Y mutation apparently changes the structure such that the mutant protein no longer effectively binds to target sequences. The same missense mutation at the corresponding position (H125Y) was identified in Tpit (TBX19). Tpit-H125Y does not bind to the consensus palindrome in EMSA and is inactive in target gene activation in a transfection assay (82).

### Repressor Activity in TBX3 Mutant Proteins

In our transient transfection assay, the DNA-binding-deficient TBX3 mutants, TBX3dm (Figures 3A and 4A) and H187Y (Figure 4A), still caused about 40–60% repression of p21-Luc. Like repression by normal TBX3, this repression was dose dependent (Figure 3A). These findings suggest that repression of p21-Luc occurs by two mechanisms. In one, TBX3 acts as an active, DNA-bound repressor; in the other, TBX3 is a co-repressor. Repression by TBX2 and TBX3 largely depends on a C-terminal repression domain (12, 35, 36). In TBX3, this repression domain was shown to be essential for the immortalization of primary embryonic fibroblasts (35). Y163fs2*, which lacks DNA binding and repression domain, did not repress p21-Luc (Figure S1 in Supplementary Material). TBX2 has been reported to act as a co-repressor on promoter constructs of two breast cancer tumor suppressor genes (NDRG1 and CST6) by binding to the transcription factor EGR1 (24, 53). Thus, there is precedence for both repression mechanisms. While TBX3dm could repress transfected p21-Luc to some extent (Figure 4A), it completely failed to repress the endogenous *p21* gene (Figure 5), indicating a higher stringency of the co-repression mechanism in the natural *p21* chromosomal context.

Repression of p21-Luc by TBX3 mutant proteins corresponded to their DNA-binding *in vitro*. H187Y with low DNA binding did not differ from the DNA-binding-deficient TBX3dm. The repression by T210delT with intermediate DNA binding was intermediate between normal TBX3 and TBX3dm, while N212delN, in which DNA binding was barely compromised, repressed nearly as strongly as normal TBX3 (Figure 4A). In contrast, all three point mutants were effective repressors of the endogenous gene indicating that, in the native chromatin context, a partly functional T-domain sufficed for full repression (Figure 5). The T-domain of T-box genes is not only required for DNA binding but also for protein–protein interactions. The interaction with many chromosomal proteins is mediated by the T-domain (84–91). Presumably, weak perturbations of the T-box still allow binding to such factors in the native chromatin context and, thus, effective gene repression by mutant TBX3 proteins with attenuated DNA binding.

### Somatic *TBX3* Mutations in Breast Cancer

Our analysis of *TBX* mutations in the ICGC data set showed that in breast cancer (BRCA-US), *TBX3* was the most frequently mutated *TBX* gene among the 16 human paralogs. This is not due to *TBX3* presenting an unusually large target size, *TBX3* being smaller than the average *TBX* gene. We also reduced the target size effect by concentrating on the ORF, which only varies about twofold among the *TBX* genes. We noted that, over the entire set of somatic cancer genome projects, two types of mutations affecting the ORF were significantly enriched in *TBX3* compared to the other *TBX* genes: frameshift mutations and in-frame deletions (Table S4 in Supplementary Material). A large part of these mutations arose in breast cancer (71 and 40%, respectively; compare Table 2 with Table S4A in Supplementary Material). Restricting the analysis to breast cancer revealed also a slight enrichment of missense mutations in *TBX3* (Table 2).

Frameshifts cause a truncation of the mutant protein downstream of the mutation site and can be expected to be all the more severe, the closer the mutations lie to the N-terminus. In breast cancer, the frameshift mutations in *TBX3* showed an extreme bias to the N-terminal half of the protein (Figure 7). With the exception of N673fs, all frameshift mutations can be expected to cause loss of function either by disruption of the DNA-binding domain or of the nuclear localization sequence (R294fs). E307fs, which causes a sequence change just downstream of the T-box, could potentially be a dominant negative mutation (5, 28, 92, 93). We demonstrated loss of function for Y163fs2*, which lacked DNA binding (Figure 2C) and failed to repress p21-Luc (Figure S1 in Supplementary Material). In the case of missense mutations, conclusion with regard to residual function of the mutant proteins cannot be drawn as easily. The enrichment for mutations in conserved residues suggests that most will lead to loss of function.

Our experimental data with the frameshift Y163fs2*, the missense H187Y, and the in-frame deletion mutant proteins (T210delT) revealed partial or complete loss of function *in vitro* (EMSA) and in cell culture (p21-Luc repression). While this loss of function is in agreement with the expectations from the statistical analysis of *TBX3* mutations in breast cancer as outlined above, it is in apparent contradiction to several previous observations on the role of TBX3 in oncogenesis. In general, it was overexpression of *TBX3*, which was found associated with oncogenic processes at the organismic, cellular, and gene regulatory level. This we outline in the following mainly for breast cancer and melanoma but similar findings were made for other types of cancer (27, 94–98).

*TBX3* was reported to be overexpressed in breast cancer cell lines and in primary cancer tissue (37, 79). An increased level of TBX3 was observed in blood plasma from patients with higher stage breast cancer (99). Similarly, increased expression of TBX3 was seen in melanoma cell lines (4) where TBX3 represses E-cadherin expression and causes increased invasiveness (6, 17, 18) and tumor formation *in vivo* (100). A critical role of TBX3 for cell migration was also demonstrated in the MCF7 breast cancer cell line (17, 101).

TBX3 expression also antagonizes replicative and oncogenic senescence by repressing *p14(ARF)*. This was demonstrated with a conditionally transformed mouse neuronal cell line (13) and with primary mouse fibroblasts (16, 35) but not yet, to our knowledge, with mammary cells.

TBX3 promotes growth of mammary epithelial cells both in cell culture (26) and in a transgenic mouse where it causes mammary hyperplasia in the absence of tumor formation (21). In human breast cancer cell lines and primary tissue, TBX3 mediates the effect of estrogen to induce the formation and expansion of cancer stem-like cells (20). The effect on proliferation is, however, dependent on cell type. In the breast cancer cell line MCF-7 and in vertical growth phase melanoma cell lines, both of which express TBX3 endogenously, it is knock-down of TBX3 that promotes proliferation (17). A similar phenomenon we observed for the TBX3 ortholog Omb in the wing imaginal disk of *Drosophila*, where Omb overexpression antagonizes proliferation in regions of high endogenous Omb and promotes proliferation in regions of low endogenous Omb (and *vice versa*). Omb overexpression leads to invasive cell motility in all regions of the disk epithelium (51).

Among the TBX3 mutants that we analyzed, only N212delN was not significantly compromised with regard to DNA binding and p21 repression, compared to normal TBX3. Rather, N212delN showed an aspect of gain-of-function: N212delN had higher transcript and protein abundance compared to normal TBX3. The N211-N212 doublet, encoded by tandem AAC codons, may be susceptible to slippage defects during replication or repair, such that N212delN may arise as a neutral mutation in cells under genomic stress.

The focus of this work was to study the impact of *TBX3* mutations as they were identified in primary breast cancers on the transcriptional properties of the protein. For the investigation of cell biological properties relevant to carcinogenesis, such as proliferation, motility, and invasive growth, the mutations will have to be studied in appropriate cancer cell lines.

## Author Contributions

Both authors designed the experiments, analyzed the experimental data, and wrote the manuscript. KF performed the experiments, GP the ICGC data base analysis. Both authors approved the final manuscript.

## Conflict of Interest Statement

The authors declare that the research was conducted in the absence of any commercial or financial relationships that could be construed as a potential conflict of interest.

## Footnotes

^1^http://www.cube-biotech.com/protocols

^2^http://imagej.nih.gov/ij/

^3^https://dcc.icgc.org, release 17
