# Supplementary material for: Putative Breast Cancer Driver Mutations in TBX3 Cause Impaired Transcriptional Repression
Source: Front Oncol. 2015 Oct 29;5:244. doi: 10.3389/fonc.2015.00244 (PMC4625211; doi:10.3389/fonc.2015.00244)
Supplement: Supplementary file 1 [file Image_1.PDF]

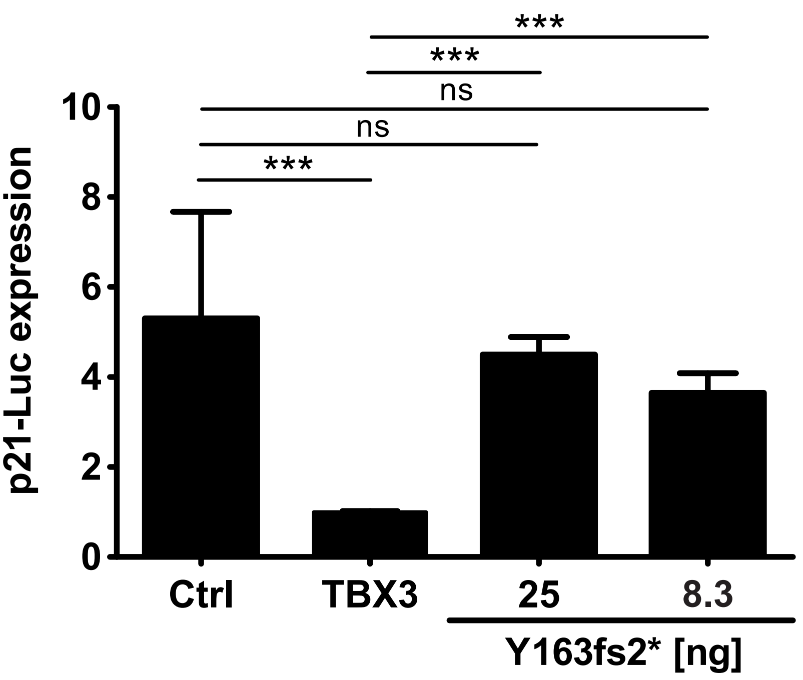

Supplementary Fig. 1
Effect of the frameshift mutant Y163fs2* on p21-Luc expression. p21-Luc expression in transfected COS-7 cells was measured at two ratios of Y163fs2 expression vector to empty vector. In all assays, a total of 25 ng of expression plasmid was used. At neither concentration was there significant repression of p21-Luc.
