# Supplementary material for: Putative Breast Cancer Driver Mutations in TBX3 Cause Impaired Transcriptional Repression
Source: Front Oncol. 2015 Oct 29;5:244. doi: 10.3389/fonc.2015.00244 (PMC4625211; doi:10.3389/fonc.2015.00244)
Supplement: Supplementary file 2 [file Table_1.PDF]

Supplementary Table 1

TBX mutations in the ICGC data base. Alphabetical list of 38 somatic cancer genome projects from the ICGC data base with mutations in TBX genes (release 17). In TBX3 (grey column), the highest number of mutations was identified in the TCGA breast cancer project (grey row).

	EOMES	T	TBR 1	T[illegible]	TBX 2	TBX 3	TBX 4	TBX 5	TBX 6	TBX 10	TBX 15	TBX 18	TBX 19	TBX 20	TBX 21	TBX 22	**total**
Acute Myeloid Leukemia - KR	0	3	0	2	2	0	3	0	0	5	1	1	0	0	5	0	**22**
Benign Liver Tumour - FR	0	0	0	0	0	0	0	0	0	0	0	0	0	0	2	0	**2**
Bladder Cancer - CN	1	1	0	0	0	1	3	2	0	2	0	0	0	1	0	0	**11**
Bladder Urothelial Cancer - TCGA, US	2	4	2	0	3	4	3	7	2	1	2	0	1	0	0	1	**32**
Brain Glioblastoma Multiforme - TCGA, US	1	2	1	1	0	0	0	4	1	0	1	2	0	1	1	3	**18**
Brain Lower Grade Glioma - TCGA, US	0	1	0	1	2	0	0	2	0	2	1	1	0	1	0	1	**12**
Breast Cancer - TCGA, US	3	4	4	2	3	27	4	8	1	5	4	7	4	4	2	10	**92**
Breast Triple Negative/Lobular Cancer - UK	0	1	0	9	1	4	6	7	1	1	9	10	5	9	1	1	**65**
Chronic Lymphocyclic Leukemia - ES	0	0	0	0	0	0	0	0	0	0	0	0	0	0	0	1	**1**
Colon Adenocarcinoma - TCGA, US	8	10	8	6	6	8	12	13	11	7	11	14	5	9	4	5	**137**
Early Onset Prostate Cancer - DE	0	1	0	0	0	0	1	0	0	0	2	1	0	0	0	1	**6**
Esophageal Adenocarcinoma - UK	6	12	10	9	1	6	18	64	1	2	69	64	16	48	9	0	**335**
Esophageal Cancer - CN	0	0	0	0	0	1	0	1	0	0	0	0	0	2	0	0	**4**
Gastric Adenocarcinoma - TCGA, US	7	15	12	5	10	8	8	19	16	10	3	20	10	3	6	8	**160**
Gastric Cancer - CN	0	1	0	0	0	0	0	0	0	0	0	0	0	1	0	1	**3**
Head and Neck Thyroid Carcinoma - TCGA, US	0	0	0	0	0	0	0	1	0	1	0	2	0	1	0	0	**5**
Kidney Renal Clear Cell Carcinoma - TCGA, US	2	0	1	0	0	0	2	0	3	0	0	1	2	0	1	0	**12**
Kidney Renal Pap. Cell Carcinoma - TCGA, US	1	0	0	0	2	2	1	0	1	1	0	0	0	0	0	0	**8**
Liver Cancer - FR	1	2	2	2	3	1	8	9	6	3	22	67	8	2	6	4	**146**
Liver Cancer - NCC, JP	7	3	7	9	4	4	7	22	3	2	10	14	7	12	5	8	**124**
Liver Cancer - RIKEN, JP	8	12	11	0	6	11	17	52	2	5	73	73	24	67	11	24	**396**
Lung Cancer - KR	0	6	1	2	7	1	0	8	3	1	7	4	2	6	0	9	**57**
Lung Squamous Cell Carcinoma - TCGA, US	4	9	5	0	6	12	1	9	2	4	2	16	5	8	5	13	**101**
Malignant Lymphoma - DE	1	2	1	1	1	7	4	11	2	1	7	6	2	3	0	11	**60**
Oral Cancer - IN	2	0	1	0	0	0	1	1	0	8	57	0	0	1	0	0	**71**
Ovarian Cancer - AU	4	6	1	12	3	10	11	34	3	8	55	43	8	20	3	14	**235**
Ovarian Serous Cystadenoca. - TCGA, US	2	0	1	1	0	0	0	1	1	0	0	0	0	0	0	0	**6**
Pancreatic Cancer - AU	12	11	10	11	8	17	20	51	5	0	0	27	14	37	12	20	**255**
Pancreatic Cancer - CA	3	4	2	3	4	6	3	16	1	1	17	8	14	6	4	3	**95**
Pancreatic Cancer Endocrine neoplasms - AU	0	1	0	1	1	2	3	4	1	0	5	8	0	7	0	2	**35**
Pediatric Brain Cancer - DE	0	0	1	0	0	0	2	4	0	0	5	10	2	2	0	3	**29**
Prostate Adenocarcinoma - CA	1	0	0	0	0	0	0	3	0	0	4	0	0	1	0	0	**9**
Prostate Adenocarcinoma - TCGA, US	2	0	0	0	0	4	0	1	0	0	0	4	1	4	0	0	**16**
Prostate Adenocarcinoma - UK	1	1	0	0	0	2	1	0	0	0	0	2	0	0	0	1	**8**
Rectum Adenocarcinoma - TCGA, US	0	1	2	1	4	2	2	0	0	2	1	1	0	2	1	3	**22**
Renal Cell Cancer - EU/FR	1	2	3	5	9	2	15	16	1	2	19	27	5	10	5	2	**124**
Skin Cutaneous melanoma - TCGA, US	12	14	15	6	8	9	12	15	11	18	38	22	15	25	8	11	**239**
Thyroid Cancer - SA	1	2	4	4	8	2	3	5	5	4	3	2		1	3	3	**50**
**total**	**93**	**131**	**105**	**93**	**102**	**153**	**171**	**390**	**83**	**96**	**428**	**457**	**150**	**294**	**94**	**163**	**3003**
