# Supplementary material for: Putative Breast Cancer Driver Mutations in TBX3 Cause Impaired Transcriptional Repression
Source: Front Oncol. 2015 Oct 29;5:244. doi: 10.3389/fonc.2015.00244 (PMC4625211; doi:10.3389/fonc.2015.00244)
Supplement: Supplementary file 3 [file Table_2.PDF]

Supplementary Table 2

Comparison of TBX3 somatic mutation rate to that in other TBX genes. The list of 38 somatic cancer genome projects from the ICGC data base with mutations in TBX genes (release 17) is sorted by decreasing TBX mutation number. Absolute mutations numbers are normalized to the total number of TBX mutation in a given cancer type. This normalized the effect of different number of tissue samples analysed and cancer-intrinsic differences in mutation rate. In the more significant top half of the table, TBX3 (grey shading) is the most frequently mutated TBX gene in breast cancer (TCGA, grey shading) but not in other types of cancer.

	EOMES	T	TBR1	T□ □	TBX2	TBX3	TBX4	TBX5	TBX6	TBX10	TBX15	TBX18	TBX19	TBX20	TBX21	TBX22	Σ	average	**total**
Liver Cancer - RIKEN, JP	2	3	2.8	0	1.5	2.8	4.3	13.1	0.5	1.3	18.4	18.4	6.1	16.9	2.8	6.1	100	6.25	**396**
Esophageal Adenocarcinoma - UK	1.8	3.6	3	2.7	0.3	1.8	5.4	19.1	0.3	0.6	20.6	19.1	4.8	14.3	2.7	0	100	6.25	**335**
Pancreatic Cancer - AU	4.7	4.3	3.9	4.3	3.1	6.7	7.8	20	2	0	0	10.6	5.5	14.5	4.7	7.8	100	6.25	**255**
Skin Cutaneous melanoma - TCGA, US	5	5.9	6.3	2.5	3.3	3.8	5	6.3	4.6	7.5	15.9	9.2	6.3	10.5	3.3	4.6	100	6.25	**239**
Ovarian Cancer - AU	1.7	2.6	0.4	5.1	1.3	4.3	4.7	14.5	1.3	3.4	23.4	18.3	3.4	8.5	1.3	6	100	6.25	**235**
Gastric Adenocarcinoma - TCGA, US	4.4	9.4	7.5	3.1	6.3	5	5	11.9	10	6.3	1.9	12.5	6.3	1.9	3.8	5	100	6.25	**160**
Liver Cancer - FR	0.7	1.4	1.4	1.4	2.1	0.7	5.5	6.2	4.1	2.1	15.1	45.9	5.5	1.4	4.1	2.7	100	6.25	**146**
Colon Adenocarcinoma - TCGA, US	5.8	7.3	5.8	4.4	4.4	5.8	8.8	9.5	8	5.1	8	10.2	3.6	6.6	2.9	3.6	100	6.25	**137**
Liver Cancer - NCC, JP	5.6	2.4	5.6	7.3	3.2	3.2	5.6	17.7	2.4	1.6	8.1	11.3	5.6	9.7	4	6.5	100	6.25	**124**
Renal Cell Cancer - EU/FR	0.8	1.6	2.4	4	7.3	1.6	12.1	12.9	0.8	1.6	15.3	21.8	4	8.1	4	1.6	100	6.25	**124**
Lung Squamous Cell Carcinoma - TCGA, US	4	8.9	5	0	5.9	11.9	1	8.9	2	4	2	15.8	5	7.9	5	12.9	100	6.25	**101**
Pancreatic Cancer - CA	3.2	4.2	2.1	3.2	4.2	6.3	3.2	16.8	1.1	1.1	17.9	8.4	14.7	6.3	4.2	3.2	100	6.25	**95**
Breast Cancer - TCGA, US	3.3	4.3	4.3	2.2	3.3	29.3	4.3	8.7	1.1	5.4	4.3	7.6	4.3	4.3	2.2	10.9	100	6.25	**92**
Oral Cancer - IN	2.8	0	1.4	0	0	0	1.4	1.4	0	11.3	80.3	0	0	1.4	0	0	100	6.25	**71**
Breast Triple Negative/Lobular Cancer - UK	0	1.5	0	13.8	1.5	6.2	9.2	10.8	1.5	1.5	13.8	15.4	7.7	13.8	1.5	1.5	100	6.25	**65**
Malignant Lymphoma - DE	1.7	3.3	1.7	1.7	1.7	11.7	6.7	18.3	3.3	1.7	11.7	10	3.3	5	0	18.3	100	6.25	**60**
Lung Cancer - KR	0	10.5	1.8	3.5	12.3	1.8	0	14	5.3	1.8	12.3	7	3.5	10.5	0	15.8	100	6.25	**57**
Thyroid Cancer - SA	2	4	8	8	16	4	6	10	10	8	6	4	0	2	6	6	100	6.25	**50**
Pancreatic Cancer Endocrine neoplasms - AU	0	2.9	0	2.9	2.9	5.7	8.6	11.4	2.9	0	14.3	22.9	0	20	0	5.7	100	6.25	**35**
Bladder Urothelial Cancer - TCGA, US	6.3	12.5	6.3	0	9.4	12.5	9.4	21.9	6.3	3.1	6.3	0	3.1	0	0	3.1	100	6.25	**32**
Pediatric Brain Cancer - DE	0	0	3.4	0	0	0	6.9	13.8	0	0	17.2	34.5	6.9	6.9	0	10.3	100	6.25	**29**
Acute Myeloid Leukemia - KR	0	13.6	0	9.1	9.1	0	13.6	0	0	22.7	4.5	4.5	0	0	22.7	0	100	6.25	**22**
Rectum Adenocarcinoma - TCGA, US	0	4.5	9.1	4.5	18.2	9.1	9.1	0	0	9.1	4.5	4.5	0	9.1	4.5	13.6	100	6.25	**22**
Brain Glioblastoma Multiforme - TCGA, US	5.6	11.1	5.6	5.6	0	0	0	22.2	5.6	0	5.6	11.1	0	5.6	5.6	16.7	100	6.25	**18**
Prostate Adenocarcinoma - TCGA, US	12.5	0	0	0	0	25	0	6.3	0	0	0	25	6.3	25	0	0	100	6.25	**16**
Brain Lower Grade Glioma - TCGA, US	0	8.3	0	8.3	16.7	0	0	16.7	0	16.7	8.3	8.3	0	8.3	0	8.3	100	6.25	**12**
Kidney Renal Clear Cell Carcinoma - TCGA, US	16.7	0	8.3	0	0	0	16.7	0	25	0	0	8.3	16.7	0	8.3	0	100	6.25	**12**
Bladder Cancer - CN	9.1	9.1	0	0	0	9.1	27.3	18.2	0	18.2	0	0	0	9.1	0	0	100	6.25	**11**
Prostate Adenocarcinoma - CA	11.1	0	0	0	0	0	0	33.3	0	0	44.4	0	0	11.1	0	0	100	6.25	**9**
Kidney Renal Pap. Cell Carcinoma - TCGA, US	12.5	0	0	0	25	25	12.5	0	12.5	12.5	0	0	0	0	0	0	100	6.25	**8**
Prostate Adenocarcinoma - UK	12.5	12.5	0	0	0	25	12.5	0	0	0	0	25	0	0	0	12.5	100	6.25	**8**
Early Onset Prostate Cancer - DE	0	16.7	0	0	0	0	16.7	0	0	0	33.3	16.7	0	0	0	16.7	100	6.25	**6**
Ovarian Serous Cystadenoca. - TCGA, US	33.3	0	16.7	16.7	0	0	0	16.7	16.7	0	0	0	0	0	0	0	100	6.25	**6**
Head and Neck Thyroid Carcinoma - TCGA, US	0	0	0	0	0	0	0	20	0	20	0	40	0	20	0	0	100	6.25	**5**
Esophageal Cancer - CN	0	0	0	0	0	25	0	25	0	0	0	0	0	50	0	0	100	6.25	**4**
Gastric Cancer - CN	0	33.3	0	0	0	0	0	0	0	0	0	0	0	33.3	0	33.3	100	6.25	**3**
Benign Liver Tumour - FR	0	0	0	0	0	0	0	0	0	0	0	0	0	0	100	0	100	6.25	**2**
Chronic Lymphocyclic Leukemia - ES	0	0	0	0	0	0	0	0	0	0	0	0	0	0	0	100	100	6.25	**1**
