# Supplementary material for: Putative Breast Cancer Driver Mutations in TBX3 Cause Impaired Transcriptional Repression
Source: Front Oncol. 2015 Oct 29;5:244. doi: 10.3389/fonc.2015.00244 (PMC4625211; doi:10.3389/fonc.2015.00244)
Supplement: Supplementary file 4 [file Table_3.PDF]

Supplementary Table 3

Position of missense mutations in TBX3 in different types of cancer. Somatic cancer genome projects from the ICGC data base in which missense mutations in TBX3 were identified are listed alphabetically. The highest number of TBX3 mutations was identified in breast cancer (TCGA, grey shading). The number of mutations in three different regions of the TBX3 proteins is listed in separate columns. There was an enrichment of missense mutations in the 184 aa T-domain (25% of 723 aa protein length) in breast cancer but also in other tumors such as melanoma.

Project Code	Project Name	N-terminus 1-103	T-domain 104-288	C-terminus 289-723	mis-sense total	positions
BLCA-CN	Bladder Cancer - CN		1		1	223
BLCA-US	Bladder Urothelial Cancer - TCGA, US		3	1	4	109, 159, 275, 412
BRCA-US	Breast Cancer - TCGA, US		4	4	8	112, 113, 187, 187, 343, 545, 630, 700
COAD-US	Colon Adenocarcinoma - TCGA, US		2	2	4	254, 284, 605, 615
ESAD-UK	Esophageal Adenocarcinoma - UK	1		1	2	28, 653
ESCA-CN	Esophageal Cancer - CN			1	1	432
KIRP-US	Kidney Renal Papillary Cell Carcinoma - TCGA, US	1			1	8
LIRI-JP	Liver Cancer - RIKEN, JP		1		1	169
LUSC-KR	Lung Cancer - KR			1	1	409
LUSC-US	Lung Squamous Cell Carcinoma - TCGA, US		2	3	5	120, 260, 302, 444, 597
OV-AU	Ovarian Cancer - AU			1	1	459
PACA-AU	Pancreatic Cancer - AU		1	2	3	223, 463, 715
PACA-CA	Pancreatic Cancer - CA		1	1	2	200, 391
PRAD-US	Prostate Adenocarcinoma - TCGA, US			3	3	595, 653, 654
READ-US	Rectum Adenocarcinoma - TCGA, US		1		1	229
SKCM-US	Skin Cutaneous melanoma - TCGA, US	1	3	1	5	42, 122, 232, 233, 291
STAD-US	Gastric Adenocarcinoma - TCGA, US		1	2	3	115, 372, 392
