# Supplementary material for: Putative Breast Cancer Driver Mutations in TBX3 Cause Impaired Transcriptional Repression
Source: Front Oncol. 2015 Oct 29;5:244. doi: 10.3389/fonc.2015.00244 (PMC4625211; doi:10.3389/fonc.2015.00244)
Supplement: Supplementary file 5 [file Table_4.PDF]

Supplementary Table 4

TBX mutations in the ICGC data base affecting the open reading frame. Mutations affecting the ORF of TBX genes are listed according to mutation type for 16 TBX genes. Data from all types of cancer and five types of ORF mutations are considered. Columns containing TBX3 data are shaded grey. (A) absolute numbers. Next to TBX18 and TBX5, TBX3 contains the highest number of ORF mutations. (B) Normalization to the number of mutations in a given TBX gene. This normalizes the data to differences in target size (cf. Table 2). Two types of mutations are enriched in TBX3: frameshift and in-frame deletions. (C) Normalization to the total number of mutations in a given mutation type across TBX genes making conspicuous the drastic enrichment of in-frame deletions in TBX3.

A) TBX ORF mutations in ICGC data set

	EOMES	T	TBR1	TBX1	TBX2	TBX3	TBX4	TBX5	TBX6	TBX10	TBX15	TBX18	TBX19	TBX20	TBX21	TBX22	Σ	Σ/16
frameshift	6	4	5	3	10	21	6	8	6	6	3	3	1	1	2	2	87	5.44
missense	37	38	34	12	29	45	28	61	39	32	50	60	23	42	22	46	598	37.38
syn	10	27	22	16	15	21	21	28	18	19	16	33	24	16	14	13	313	19.56
stop gained	3	3	4	0	2	4	3	1	1	2	2	3	1	3	1	1	34	2.13
in-frame del	1	0	0	0	0	5	0	0	0	0	0	0	0	0	0	0	6	0.38
**sum**	**57**	**72**	**65**	**31**	**56**	**96**	**58**	**98**	**64**	**59**	**71**	**99**	**49**	**62**	**39**	**62**	**1038**	

B) TBX ORF mutations in ICGC data set (as percentage of sum of mutations in a given TBX gene)

	EOMES	T	TBR1	TBX1	TBX2	TBX3	TBX4	TBX5	TBX6	TBX10	TBX15	TBX18	TBX19	TBX20	TBX21	TBX22	Σ/16
frameshift	10.53	5.56	7.69	9.68	17.86	21.88	10.34	8.16	9.38	10.17	5.87	2.97	2.04	1.61	5.13	3.23	8.11
missense	64.91	52.78	52.31	38.71	51.79	46.88	48.28	62.24	60.94	54.24	97.80	59.41	46.94	67.74	56.41	74.19	56.29
syn	17.54	37.50	33.85	51.61	26.79	21.88	36.21	28.57	28.13	32.20	31.30	32.68	48.98	25.81	35.90	20.97	31.13
stop gained	5.26	4.17	6.15	0.00	3.57	4.17	5.17	1.02	1.56	3.39	3.92	2.97	2.04	4.84	2.56	1.61	3.19
in-frame del	1.75	0.00	0.00	0.00	0.00	5.21	0.00	0.00	0.00	0.00	0.00	0.00	0.00	0.00	0.00	0.00	0.43
**sum**	**100.00**	**100.00**	**100.00**	**100.00**	**100.00**	**100.00**	**100.00**	**100.00**	**100.00**	**100.00**	**100.00**	**100.00**	**100.00**	**100.00**	**100.00**	**100.00**	**100.00**

C) TBX ORF mutations in ICGC data set (as percentage of total mutations of a given mutation type)

	EOMES	T	TBR1	TBX1	TBX2	TBX3	TBX4	TBX5	TBX6	TBX10	TBX15	TBX18	TBX19	TBX20	TBX21	TBX22	Σ
frameshift	6.90	4.60	5.75	3.45	11.49	24.14	6.90	9.20	6.90	6.90	3.45	3.45	1.15	1.15	2.30	2.30	100.00
missense	6.19	6.35	5.69	2.01	4.85	7.53	4.68	10.20	6.52	5.35	8.36	10.03	3.85	7.02	3.68	7.69	100.00
syn	3.19	8.63	7.03	5.11	4.79	6.71	6.71	8.95	5.75	6.07	5.11	10.54	7.67	5.11	4.47	4.15	100.00
stop gained	8.82	8.82	11.76	0.00	5.88	11.76	8.82	2.94	2.94	5.88	5.88	8.82	2.94	8.82	2.94	2.94	100.00
in-frame del	16.67	0.00	0.00	0.00	0.00	83.33	0.00	0.00	0.00	0.00	0.00	0.00	0.00	0.00	0.00	0.00	100.00
**percentage of total TBX ORF mutations**	**5.49**	**6.94**	**6.26**	**2.99**	**5.39**	**9.25**	**5.59**	**9.44**	**6.17**	**5.68**	**6.84**	**9.54**	**4.72**	**5.97**	**3.76**	**5.97**	**100.00**
