# Supplementary material for: Putative Breast Cancer Driver Mutations in TBX3 Cause Impaired Transcriptional Repression
Source: Front Oncol. 2015 Oct 29;5:244. doi: 10.3389/fonc.2015.00244 (PMC4625211; doi:10.3389/fonc.2015.00244)
Supplement: Supplementary file 6 [file Table_5.PDF]

Supplementary Table 5
Primer sequences.

primer number	primer sequence
1323	ACACAAAGCTTCTGACTTCGGCAGCTGCT
1379	GTCATTACCAAGTCGGCTAGCCGAATGTTTCCTCCATTTAAAG
1380	CTTTAAATGGAGGAAACATTCGGCTAGCCGACTTGGTAATGAC
1560	CCACAAACTGAAACTCACCAACATTTCAGACAAACATGG
1561	CCATGTTTGTCTGAAATGTTGGTGAGTTTCAGTTTGTGG
1562	GCTGCTGATGACTGTCGTTTATAAATTTCACAATTCTCGG
1563	CCGAGAATTGTGAAATTTATAAACGACAGTCATCAGCAGC
1564	CGTCACTTTCCACAAACTGAAACTCAACAACATTTCAGACAAACATGG
1565	CCATGTTTGTCTGAAATGTTGTTGAGTTTCAGTTTGTGGAAAGTGACG
1760	TCTTCCAGCCTTCCTTCCTG
1761	CAATGCCAGGGTACATGGTG
1772	CAGCAGAGGAAGACCATGTG
1773	GTGGTAGAAATCTGTCATGCTG
1784	CCAAAGAGGATGTACATTTACCCGGACAGCCCCGCTACTG
1785	CAGTAGCGGGGCTGTCCGGGTAAATGTACATCCTCTTTGG
1808	ACACAGGTACCGCTTGGGCAGCAGGCTGT
1879	CTCAGAGGATCCATGAGCCTCTCCATGAGAGATCCG
1880	TCTGAGGTCGACCTATCATTCACTGGAGGACTCATCAGAGG
1924	TCCCAAGTGATCACGCTACG
1925	CTGGTATGCAGTCACAGCGA
